# Remote data possession checking scheme with supporting efficient group user authority management for shared cloud data

**DOI:** 10.1038/s41598-023-40682-9

**Published:** 2023-08-21

**Authors:** Yilin Yuan, Zhenzhen Zhang

**Affiliations:** College of Information Engineering, Beijing Institute of Graphic Communication, Beijing, 102600 China

**Keywords:** Engineering, Mathematics and computing

## Abstract

Under the shared big data environment, the existing shared data auditing schemes rarely consider the authorization management of group users. Thus, in this paper, we propose a novel remote shared data checking possession scheme that support group authority management. To implement group user rights management, we firstly introduce a trusted entity group manager. To assist the group manager in authorization management, we formalize a new algebraic structure operator named authorization invisible authenticator (AIA). Meanwhile, we provide a basic AIA scheme for general security scenarios and a standard AIA scheme suitable for high-level security scenarios respectively. The standard AIA scheme can fully meet the needs of the group manager to safely perform rights management work. It is composed of the User Information Table (UIT) and the basic AIA scheme, which has higher security and is applicable to a wider range of scenarios. By distributing AIA through the standard AIA scheme, the group manager can easily carry out authority management, including enrolling, revoking, updating. After solving the problem of authorization management, the detailed design of the scheme based on identity-based encryption (IBE) is given. Furthermore, the security analysis and performance evaluation demonstrate that the scheme is safe and feasible.

## Introduction

As cloud computing becomes more accepted by the public, cloud storage is also widely used. Cloud Service Provider (CSP) supply users with large-capacity and reasonably priced storage services. Users can choose different types of cloud storage services to store outsourced data according to their needs. Cloud storage has many advantages, such as pay on-demand, location independence, and off-site management, which makes it more popular. From an objective point of view, because users no longer have absolute control over outsourced data, the security and integrity of data on the cloud is facing huge risks. Once an error occurs, it will inevitably cause serious losses for users.

With the assistance of data sharing services, users can share data stored on the CSP with other users in the group. Using sharing data, users can access data without repeated storage, which not only relieves the local storage burden, but also reduces the communication overhead caused by multiple interactions with the CSP. Therefore, group data sharing services are very popular with users. However, the existing shared data integrity verification schemes consider such issues as how to share data within a group, and how to verify the integrity of the shared data after the user who possessed the shared data is revoked, and rarely consider group user rights management. To be exact, within a group, only users with valid permissions can access the shared data and can access freely. But in practical, the authorization management of group users is cumbersome and difficult, and there may exist some difficulties: (1) How to make the users who apply to enroll the group become valid group users? (2) How to revoke the access rights to the shared data of users who withdraw the group? Correspondingly, if the users have uploaded shared data into the group, how should the ownership of the data be reclaimed? (3) In addition, how to deal with the shared data integrity verification problem also needs to be considered. Thus, to solve the above-mentioned challenge, this paper conducts a detailed discussion on the permission management of group users under the shared data integrity checking problem.

### Contribution

To share cloud data, we construct a remote data possession checking scheme with supporting efficient group user authority management. The contributions are summarized as follows:

1. To deal with the issue of rights management of users within a group, in our proposed scheme, we introduce a trusted entity—group manager. The inclusion of a group manager has not only simplified the complexity of group rights management but also realizes uniformity within the group.
2. To assist the group manager in authority management, we further propose a novel algebraic structure operator called authorization invisible authenticator (AIA). AIA is an operator generated by the group manager and available to the group users but with a transparent structure. Then, we provide detailed algorithms of the basic AIA scheme. However, we found that the basic AIA scheme cannot fully meet the needs of group manager to safely perform rights management. Therefore, a standard AIA scheme jointly constructed by the basic AIA scheme and the user information table (UIT) is developed, which has advanced security and is applicable to a wider range of scenarios. The security of the standard AIA scheme has been rigorously proved from four aspects: correctness, invisibility, unforgeability and non-malleability. The group manager distribute AIAs by means of standard AIA schemes, and only the group users possess valid AIAs are regrade as valid group users and can freely access shared data within the group. Meanwhile, the group manager can easily carry out authority management work including group user joining, revoking, and updating.
3. Finally, on the basis of solving the problem of authorization management, the shared data integrity verification scheme is further provided. The design details are as follows: The scheme is constructed based on identity-based encryption (IBE), which significantly reduces the overhead of certificate processing caused by Public Key Infrastructure (PKI) distributing public and private keys. Our proposed scheme can ensure key correctness, AIA correctness and audit correctness. Moreover, under the given random oracle model, the soundness based on the Computational Diffie-Hellman Assumption and the Discrete Log Assumption is rigorously proved. Finally, the performance evaluation confirms that the scheme is feasible and effective.

### Related work

The traditional remote data possession checking method requires users to download all their data stored in the cloud locally, and then perform checking. For users with low-capacity equipment or low computing capacity, the cost is fatal. In 2007, with the successive schemes of Provable Data Possession (PDP)^1^ and Proof of Retrievability (PoR)^2^, the research on data integrity verification was pushed to an unprecedented climax. The PDP scheme is based on homomorphic authenticators and random sampling strategy and uses RSA to design the tags set. The PoR scheme is implemented in bilinear pairing, which not only provides integrity verification, but also enables data retrieval. In addition, based on PoR, Shacham et al.^3^ proposed two schemes which can complete the public verifiability and private verifiability using BLS signature and pseudorandom function respectively. Based on PDP or PoR scheme, more valuable proposals are proposed^4–12^. But unfortunately, all the schemes mentioned above are implemented by Public Key Infrastructure (PKI). Public key cryptography based on PKI distribution keys has been widely used since its introduction. However, due to the existence of certificates, a series of costs such as generation, forwarding, and renewal are inevitably increased. Using the user's public identifier, such as the email address, as an encryption key can solve a series of problems caused by public key certificates^13,14^. Based on Identity- Based Encryption (IBE), the first public data integrity auditing scheme was proposed^15^. And subsequently, more interesting schemes based on IBE construction were proposed^16–23^.

The group data sharing services under the integrity verification problem has been deeply studied in recent years. To address the issue of integrity verification of shared cloud data, Wang et al. proposed three different schemes^24–26^. Konx^24^ focused on the privacy protection within the group and utilized group signatures to construct homomorphic authenticators to complete data checking. Different from Konx, Oruta^25^ considered using ring signatures to achieve mathematical design. Panda^26^ explored how to employ proxy re-signatures to realize group user revocation. Yuan et al.^27^ discussed a public integrity auditing scheme that can support multiple users to modify shared data. Tian et al.^28^ given a comprehensive group management integrity auditing scheme which considers privacy protection, dynamics, and traceability of shared data. To support user revocation effectively, Zhang et al.^29^ explored a new strategy for key generation phase and a technique for private key update. By constructing a homomorphic verifiable group signature in the privacy-aware public auditing mechanism. Fu et al.^30^ proposed a group shared data checking scheme which can prevent sensitive information from leaking to third-party auditors. Considering the collusion resistant attack, Luo et al.^31^ provided the scheme to realize public auditing and user revocation. In a group that shares cloud data, user revocation is a hard assignment. Although scheme^24^ claims to enable user revocation, the revocation process is akin to re-running all algorithms, incurring exorbitant costs. Scheme^25,28^ primarily focus on discussing group functionality issues, such as batch auditing, identity tracing, and others, while scheme^30^ addresses privacy concerns related to shared data within the group. None of these three schemes discuss user revocation. On the other hand, scheme^26,27,29,31^ do delve into the issue of user revocation, but the cost of revocation is almost directly proportional to the number of data blocks. Furthermore, none of the aforementioned schemes mention group user authorization management.

### Paper organization

The rest of the paper is organized as follows. In Section "Preliminaries", we present the preliminaries. And in Section "System components", we present the system components. A new algebraic structure operator named authorization invisible authenticator is explained in Section "Authorization invisible authenticator". The detailed description of the proposed scheme is elaborated in Section "The proposed scheme". Section "Security analysis" shows the security analysis. The performance evaluation is carried out in Section "Performance evaluation". Section "Conclusion" is the conclusion of this paper.

## Preliminaries

In this section, we describe the system model, design goals, notations, and cryptographic knowledge.

### System model

**Figure 1:**
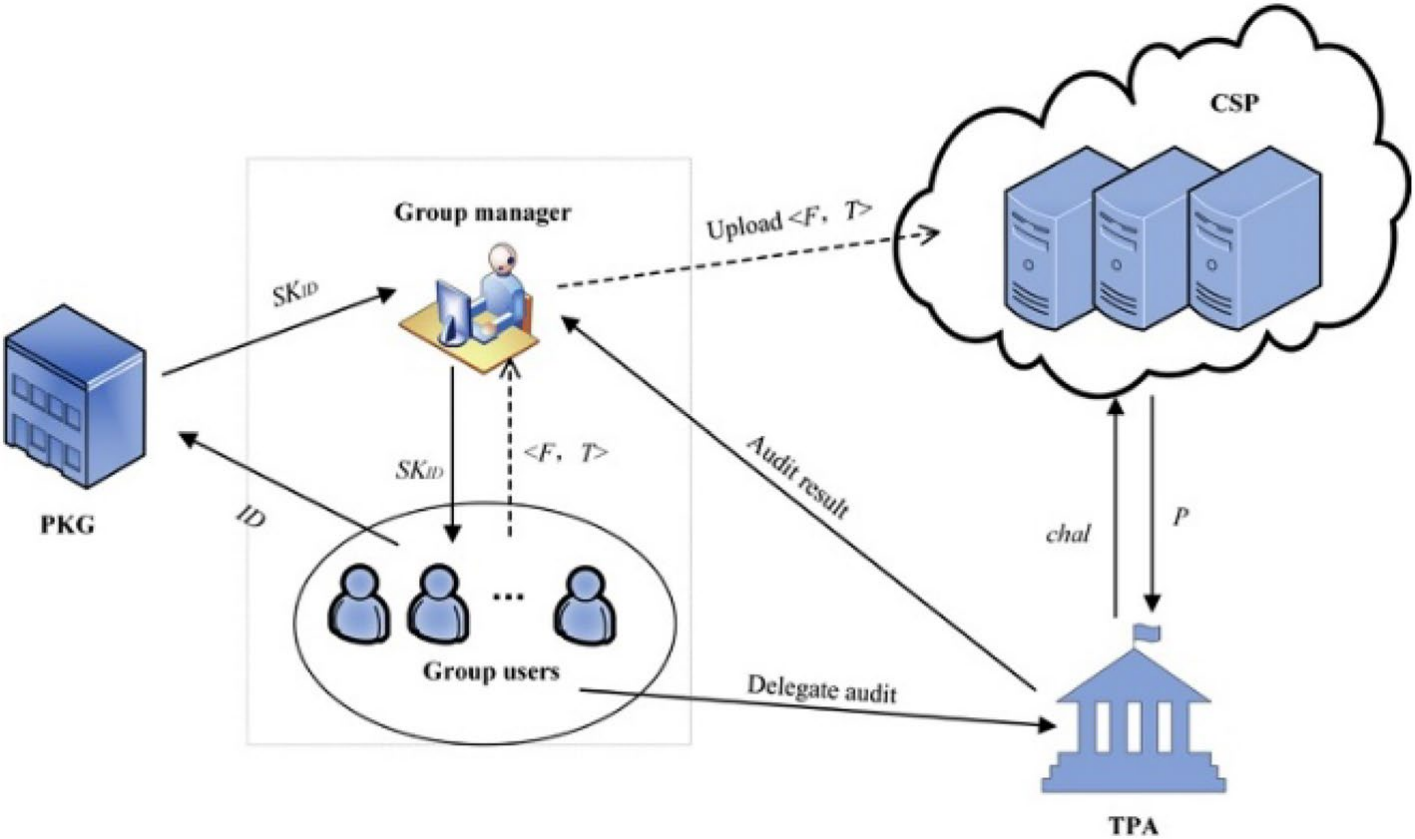
The system model diagram.

The system consists of five entities, and the model is shown in Fig. 1.

1. Group Manager: the trusted entity who performs authority management work. The group manager opens the access authorizations for the users who apply to enroll the group, and withdraws all rights for the users who leave the group. Moreover, the public audit results of shared data are also recorded by the group manager, not the creator of the shared data.
2. Group Users: when a user successfully enrolls the group, he becomes a group user, who can upload his own data to the CSP, mark it as shared data, and access the shared data of other users in the group. All unrevoked group users can upload shared data to the CSP and share it with other group users. For a revoked group user, the group manager will deprive his permission to view other shared data, but the shared files that he has uploaded to the group are still saved in the group. To be precise, once a group user uploads the file and shares it with other group users, then the file will be regarded as a group shared file. In addition, the ownership of the shared file of the revoked group user will be transferred to the group manager. Before the shared file is modified, the revoked user still knows the content of the shared file but has no right to modify it.
3. Cloud Service Provider (CSP): an untrusted entity that provides public cloud storage service.
4. Private Key Generator (PKG): the trusted entity who generates the private key for the group users.
5. Third-Party Auditor (TPA): the trusted entity who is responsible for performing remote data checking for the shared data uploaded to the CSP after being authorized by the group users. When the TPA wishes to check the integrity of the shared data, it first sends an integrity challenge to the CSP. Then the TPA checks the correctness of the audit proof returned by the CSP and records the results. The audit results will be feedback to the group manager on a regular basis.

#### *Remark 1*

The private key generation process is described in detail in Section "The proposed scheme".

#### *Remark 2*

The responsibilities of the group manager include the four parts: (a) Grant permissions to the users who apply to join the group. (b) Deprive permissions to the users who leave the group. Note that even if the user opts out of the group, he still has the right to view the shared files he uploaded, but he does not have the right to modify it. (c) Perform uploading of shared data on behalf of group users. d) Record the audit results of the shared data integrity verification.

### Design goals

A remote data possession checking scheme with supporting efficient group user authority management should achieve following goals:

(1) **Correctness:** The correctness consists of private key correctness, AIA correctness and audit correctness.

(a) **Private key correctness**: The private key generated by the PKG will only be accepted by the group user after passing the correctness verification of the group manager.

(b) **AIA correctness**: A valid AIA $\Phi$ can be passed the verification of the group manager.

(c) **Audit correctness:** If the CSP stores all the cloud data of the user, in the public auditing phase, the audit proof generated by the CSP can be verified by the TPA.

(2) **Audit soundness:** If the CSP does not possess user’s intact data, it cannot pass the TPA’s verification.

(3) **Secure authority management**: The group manager can safely and efficiently perform authority management work, and any user joining/leaving the group will not affect other users.

(4) **Efficient revocation**: When one user applies for revocation, only the group manager needs to perform the operation with lower computational overhead. In other words, the cost of user revocation is independent of the number of the revoked users.

(5) **Public auditing**: Under the authorization of the group users, the TPA performs remote data integrity verification for the shared data, and periodically returns the audit results to the group manager.

## Notations

We give the notations used in the description of our scheme in Table 1.

**Table 1:** Notations and descriptions.

Notation	Meaning	Notation	Meaning
*p*	One large prime	*G*_1_, *G*_2_	Multiplicative cyclic groups
*g*	A generator of group *G*_1_	*e*	A bilinear map: $e:G_{1} \times G_{1} \to G_{2}$
$Z_{P}^{*}$	A prime field with non-zero elements	$H,H_{1} ,H_{2}$	Cryptographic hash function
*f*	Pseudo-random function	$\pi$	Pseudo-random permutation
*pp*	The system public parameter	*ID*	The group user’s identity
*SK*_*ID*_	The group user’s private key	$msk_{ID} ,mpk_{ID}$	The master secret key and master public key
*F*	The user’s shared file	*n*	The number of blocks
$\sigma_{i}$	The *i*-th block’s tag, $1 \le i \le n$	*UN*	The number of group users

### Cryptographic knowledge

**(1) Bilinear maps**

Let $G_{1} ,G_{2}$ are multiplicative cyclic group with the order *p*, *g* is a generator of $G_{1}$. A bilinear map $e:G_{1} \times G_{1} \to G_{2}$ satisfies the following properties:

- Bilinearity: $$\forall u,v \in G_{1}$$ and $$\forall a,b \in Z_{P}^{*}$$, $$e(u^{a} ,v^{b} ) =$$$$e(u,v)^{ab}$$;
- Non-degeneracy: $$e(g_{1} ,g_{2} ) \ne 1$$;
- Computable: there is an efficient algorithm to calculate *e*.

**(2) Security assumptions**

**Computational Diffie-Hellman assumption**. For unknown $\forall a,b \in Z_{P}^{*}$, given g, $g^{a}$ and $g^{b}$ as input, output $g^{ab} \in G_{1}$.

#### **Definition 1**

(*CDH assumption*). The advantage of a *PPT* (probabilistic polynomial time) algorithm $\mathcal{A}$ in solving the CDH problem in $G_{1}$ defined below is negligible:

$$ AdvCDH_{\mathcal{A}} = \Pr [\mathcal{A}(g,g^{a} ,g^{b} ) = g^{ab} :a,b\mathop \leftarrow \limits^{R} Z_{P}^{*} ] $$

**Discrete logarithm assumption**. For unknown $x \in Z_{P}^{*}$, given g and $g^{x}$ as input, output *x*.

#### **Definition 2**

(*DL assumption*). The advantage of a *PPT* (probabilistic polynomial time) algorithm $\mathcal{A}$ in solving the DL problem in *G*_*1*_ defined below is negligible:

$$ AdvDL_{\mathcal{A}} = \Pr \left[ {\mathcal{A}(g,g^{x} ) = x:x\mathop \leftarrow \limits^{R} Z_{P}^{*} } \right] $$

## System components

In this section, we describe the system components of the proposed scheme. A remote data possession checking scheme with supporting efficient group user authority management consists of eight algorithms: **Setup**, **AuthForUser**, **KeyGen**, **TagGen**, **UpdateForUsers**, **Challenge**, **ProofGen**, **ProofVerify**. Each algorithm is described as follows:

***Setup*** $(1^{k} ) \to (pp,mpk,msk)$ is the “Setup” algorithm run by the PKG. It takes the security parameter *k* as input, and outputs the system public parameter *pp*, master public key *mpk* and master secret key *msk*.

***AuthForUser*** is the “Authorized for Group User” algorithm which consists of a series of algorithms. It takes the *pp* as input, and outputs an AIA for the group user.

***KeyGen*** $(pp,mpk,msk,ID) \to SK_{ID}$ is the “Private Key Generation” algorithm run by the PKG. It takes the *pp*, user’s identifier *ID*, *msk* and *mpk* as input, and outputs user’s private key *SK*_*ID*_.

***TagGen*** $(F,pp,ID,SK_{ID} ) \to T$ is the “Tags Set Generation” algorithm run by the group user. It takes *F*, *pp*, *ID* and *SK*_*ID*_ as input, and outputs the tags set T. Then, the user uploads <*F*, *T*> to the group manager. Subsequently, the group manager performs the data upload work.

***UpdateForUsers*** is the “Update AIA for The Rest of Group Users” algorithm run by the group manager. When one user is revoked from the group, the group manager runs this algorithm.

***Challenge*** algorithm run by the TPA that is to generate the integrity challenge *chal*.

***ProofGen*** $(chal,F,T) \to P$ is the “Proof Generation” algorithm run by the CSP. It takes the *chal*, *F* and *T* as input, and outputs the audit proof P.

***ProofVerify*** $(pp,chal,P) \to 0/1$ is the “Proof Verification” algorithm run by the TPA. It takes *pp*, *chal* and *P* as input, and outputs “0/1”; “1” indicates that the shared data stored in the CSP is intact; otherwise, it is not.

## Authorization invisible authenticator

In this section, we construct a novel algebraic structure operator to assist the group manager in conducting rights management. We first provide the details of the basic AIA scheme, then analysis its shortcomings and propose a user information table. The improved scheme named standard AIA scheme which is jointly composed of the basic AIA scheme and user information table, and its details and security proof are given.

### Overview of AIA

When a new user applies to enroll the group, the group manager performs authority management and authorized him to become a legal group user. Correspondingly, when a group user chooses to withdraw the group, the group manager should revoke all their permissions, including the right to modify the shared data they have uploaded, and the access right to view other shared data. Thus, we construct a novel algebraic structure operator named authorization invisible authenticator to facilitate the group manager to deal with permission management issues. For simplify, we call it AIA. Any valid AIA should meet the following requirements:

**Invisibility**: AIA is distributed to the group users by the group manager, and any user possess valid AIA is regarded as users in the group. AIA can only be used by the group users, but the algebraic structure is invisible to the group users; that is, AIA cannot be inferred.

**Unforgeability**: The group users cannot forge a valid AIA. The unforgeability of AIA determines that any unauthorized user cannot view the shared data in the group.

**Non-malleability**: Given two AIA $\Phi_{1} ,\Phi_{2} \in Z_{P}^{*}$, and two values $a,b \in Z_{P}^{*}$. Any valid AIA cannot be computed by linearly aggregating $\Phi = a\Phi_{1} + b\Phi_{2} \in Z_{P}^{*}$, except with negligible probability. The non-malleability of AIA ensures that no two unauthorized users can collude to forge an invalid AIA.

### Basic AIA scheme

To assist the group manager in performing authority management, we propose a basic AIA scheme which consists of four algorithms:

1. **Init** $(1^{k} \to pp)$: This is “Init” algorithm to initialize public parameters. It takes the security parameter *k* as input, and outputs the public parameter *pp* (public parameter *pp* mentioned here are same as those initialized in the “**Setup**” phase in Section "The proposed scheme").
2. **ApplyForAuth** $(pp \to rm)$: This is “Apply for Authorization” algorithm run by the group user. When a new user applies to join the group, he needs to ask for the group manager to authorize him to become a group user. It takes the *pp* as input, and outputs a requirement message *rm*. Note that the basic AIA scheme stipulates that user only send *rm* once when they register as a group user.
3. **AuthGen** $(rm,pp \to \Phi )$: This is “AIA Generation” algorithm run by the group manager. It takes the *rm* and *pp* as input, and outputs an AIA $\Phi$ for the new user.
4. **AuthUpdate**: This is “AIA Update” algorithm run by the group manager. When the users leave the group, the group manager runs this algorithm to redistribute the AIAs for the remaining group users.

### User information table (UIT)

The basic AIA scheme can meet the needs of most schemes for authorization management. However, in some scenarios, the basic AIA scheme may exist two security threats: (1) Despite in the **ApplyForAuth** algorithm, the basic AIA scheme stipulates that the user sends *rm* to the group manager only once; However, there may be malicious group users who, after sending *rm* to the group manager and obtaining AIA, continue to repeatedly send *rm* to the group manager and expect to get more AIA. And its ultimate goal is to obtain multiple AIAs and try to derive the mathematical structure of the AIA in the hope of forging a reliable AIA. (2) There may be a curious group user trying to apply for AIA to the group manager using another valid *rm*. Considering the above potential security risks, it is necessary for the group manager to preserve a User Information Table (UIT) locally. With the help of UIT, the security of the basic AIA scheme can be further improved.

The group manager maintains a local UIT to record the information of every group user. The UIT contains five columns, and its structure is illustrated in Table 2. *UN* represents the number of current group users. *ID* is the group user’s identifier. *rm* is the requirement message sent to the group manager when the user joins the group. AIA is a legal access authenticator distributed to the users by the group manager. *SK*_*ID*_ is the private key of the group user obtained from the PKG (The usage of UIT will be shown in Section "The proposed scheme").

**Table 2:** The structure of the UIT.

*UN*	*ID*	*rm*	AIA	*SK*_*ID*_
1	*ID*_1_	$\left( {z_{1}^{{r_{1} }} ,ID_{1} } \right)$	AIA_1_	$SK_{{ID_{1} }}$
2	*ID*_2_	$\left( {z_{2}^{{r_{2} }} ,ID_{2} } \right)$	AIA_2_	$SK_{{ID_{2} }}$
…	…	…	…	…

There are advantages of adding the UIT: (1) The UIT records the matching relationship between *rm* and the AIA. In the **ApplyForAuth** phase, the group manager will only respond to the first *rm* sent by the group user. In addition, when a user is withdrawn from the group, the group manager will immediately delete the user’s information from the UIT and update the AIAs of the remaining group users. (2) Only when a user's *ID*, *rm*, AIA and *SK*_*ID*_ form a one-to-one correspondence, that is, they are all in the same row of UIT, this user is regarded as a valid group user. Invalid group users will be removed from the group by the group mana-ger.

### Standard AIA scheme

**Figure 2:**
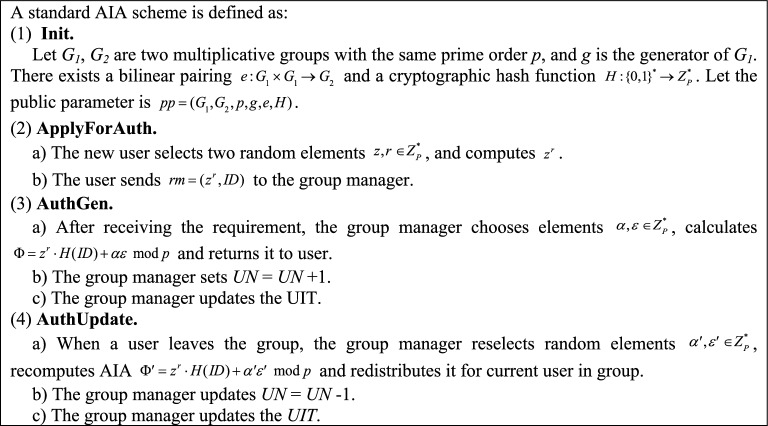
The process of standard AIA scheme.

The standard AIA scheme is composed of the basic AIA scheme and the UIT, as shown in Fig. 2.

#### *Remark 3*

In the standard AIA scheme, the group manager will reassign AIAs to the remaining group users if and only after the users leave the group.

#### *Remark 4*

Note that in the standard AIA scheme we proposed, the cost of user revocation in a group is irrelevant to the shared data, but only with the number of the group users.

### Security of AIA scheme

A standard AIA scheme should satisfy the above properties. We prove it to be secure in terms of correctness, invisibility, unforgeability and non-malleability.

#### **Theorem 1**

(Correctness) *Given a legal AIA* $\Phi$, *the group manager can verify its correctness*.

#### *Proof*

The group manager can parse the AIA $\Phi$ through the following derivation and further to verify its correctness.

$$ g^{\Phi } = g^{{z^{r} \cdot H(ID) + \alpha \varepsilon }} = g^{{z^{r} \cdot H(ID)}} \cdot g^{\alpha \varepsilon } = (g^{{z^{r} }} )^{H(ID)} \cdot g^{\alpha \varepsilon } $$

#### **Theorem 2**

(Invisibility). *The AIA's algebraic structure is invisible to the group users*.

#### *Proof*

Since the AIA’s algebraic structure is confidential and transparent, AIA is invisible to any group users.

#### **Theorem 3**

(Unforgeability). *For any adversary* $\mathcal{A}$, *it is computationally infeasible to successfully forge AIA*.

#### *Proof*

Assume $\Phi_{1}$ is a legal AIA, and adversary $\mathcal{A}$ has successfully forged an AIA denoted as $\Phi_{2}$. We give a brief proof as follows:

The valid AIA is $\Phi_{1} = z^{r} \cdot H(ID) + \alpha \varepsilon \, \bmod p$, and we have:

1 $$ g^{{\Phi_{1} }} = g^{{z^{r} \cdot H(ID) + \alpha \varepsilon }} $$

The forged AIA is $\Phi_{2} = z^{r} \cdot H(ID) + \alpha^{\prime}\varepsilon^{\prime} \, \bmod p$, and we also have:

2 $$ g^{{\Phi_{2} }} = g^{{z^{r} \cdot H(ID) + \alpha^{\prime}\varepsilon^{\prime}}} $$

Divide (1) by (2), and obtain:

$$ {{g^{{\Phi_{1} }} } \mathord{\left/ {\vphantom {{g^{{\Phi_{1} }} } {g^{{\Phi_{2} }} }}} \right. \kern-0pt} {g^{{\Phi_{2} }} }} = {{g^{{z^{r} \cdot H(ID) + \alpha \varepsilon }} } \mathord{\left/ {\vphantom {{g^{{z^{r} \cdot H(ID) + \alpha \varepsilon }} } {g^{{z^{r} \cdot H(ID) + \alpha^{\prime}\varepsilon^{\prime}}} }}} \right. \kern-0pt} {g^{{z^{r} \cdot H(ID) + \alpha^{\prime}\varepsilon^{\prime}}} }} = {{g^{\alpha \varepsilon } } \mathord{\left/ {\vphantom {{g^{\alpha \varepsilon } } {g^{{\alpha^{\prime}\varepsilon^{\prime}}} }}} \right. \kern-0pt} {g^{{\alpha^{\prime}\varepsilon^{\prime}}} }} $$

According to the above assumptions, we can know $1 = {{g^{\alpha \varepsilon } } \mathord{\left/ {\vphantom {{g^{\alpha \varepsilon } } {g^{{\alpha^{\prime}\varepsilon^{\prime}}} }}} \right. \kern-0pt} {g^{{\alpha^{\prime}\varepsilon^{\prime}}} }} = g^{{\alpha \varepsilon - \alpha^{\prime}\varepsilon^{\prime}}}$, and further we get $\alpha \varepsilon = \alpha^{\prime}\varepsilon^{\prime} = 0\bmod p$. Here $\alpha ,\varepsilon \in Z_{P}^{*}$ are two large prime selected randomly by the group manager. Obviously, the probability of $\alpha \varepsilon = \alpha^{\prime}\varepsilon^{\prime} = 0\bmod p$ is $p \times p = p^{2}$ which is negligible.

Therefore, it can be inferred that the probability that the adversary $\mathcal{A}$ successfully forges a valid AIA is negligible.

#### **Theorem 4**

(Non-malleability) For any two adversaries $\mathcal{A}_{1} , \, \mathcal{A}_{2}$, the AIA cannot be forged in a linearly aggregated manner.

#### *Proof*

Suppose that two adversaries $\mathcal{A}_{1} , \, \mathcal{A}_{2}$ possess *rm*_1_, *rm*_2_ certified by the group users, and haven got AIA $\Phi_{1} ,\Phi_{2}$ respectively. Given a valid AIA denoted as $\Phi = z^{r} \cdot H(ID) + \alpha \varepsilon \, \bmod p$. The non-malleability of the AIA scheme can be proved by the following:

Two adversaries $\mathcal{A}_{1} , \, \mathcal{A}_{2}$ respectively choose random values $a,b \in Z_{P}^{*}$, and they try to linearly aggregate AIA $\Phi^{\prime} = a\Phi_{1} + b\Phi_{2} \in Z_{P}^{*}$ to obtain $\Phi$. But in fact, there is no way to infer a valid AIA $\Phi$, the reason is as follows:

The algebraic structure of valid AIA is $\Phi = z^{r} \cdot H(ID) + \alpha \varepsilon \, \bmod p$. And the algebraic structure of the aggregated AIA is

$$ \begin{aligned} \Phi^{\prime } & = a\Phi_{1} + b\Phi_{2} \\ & = a\left( {z_{1}^{{r_{1} }} \cdot H(ID_{1} ) + \alpha \varepsilon \, \bmod p) + b(z_{2}^{{r_{2} }} \cdot H(ID_{2} ) + \alpha \varepsilon \, \bmod p} \right) \\ & = \left( {az_{1}^{{r_{1} }} \cdot H(ID_{1} ) + bz_{2}^{{r_{2} }} \cdot H(ID_{2} )} \right) + (a + b) \cdot \alpha \varepsilon \, \bmod p \ne z^{r} \cdot H(ID) + \alpha \varepsilon \, \bmod p = \Phi \\ \end{aligned} $$

Obviously, $\Phi^{\prime} \ne \Phi$. So, the valid AIA $\Phi$ cannot be forged by two adversaries $\mathcal{A}_{1} , \, \mathcal{A}_{2}$ through linear aggregation.

## The proposed scheme

A remote data possession checking scheme with supporting efficient group user authority management consists of eight algorithms, which are introduced in detail in this section.

**(1) Setup**

- The PKG chooses two multiplicative cyclic groups *G*_*1*_ and *G*_*2*_ of prime order *p*, and *g* is a generator of *G*_*1*_. The PKG selects cryptographic hash function $H,H_{1} :\{ 0,1\}^{*} \to Z_{P}^{*} , \, H_{2} :\{ 0,1\}^{*} \to G_{1}$, the bilinear map $e:G_{1} \times G_{1} \to G_{2}$, pseudo-random function (PRF) $f:Z_{P}^{*} \times \{ 1,2,...,n\} \to Z_{P}^{*}$, pseudo-random permutation (PRP) $\pi :Z_{P}^{*} \times \{ 1,2,...,n\} \to \{ 1,2,...,n\}$.
- The PKG selects elements $x_{ID} \in Z_{P}^{*}$, computes the master secret key $msk_{ID} = x_{ID}$ and master public key $mpk_{ID} = g^{{x_{ID} }}$.
- The PKG randomly picks values $\mu \in G_{1}$.
- The PKG publishes the system public parameter $pp = (G_{1} ,G_{2} ,p,e,g,f,\pi ,H,H_{1} ,H_{2} ,\mu ,mpk_{ID} )$ and holds the master secret key $msk_{ID} = x_{ID}$ private.

**(2) AuthForUser**

- The new user enrolled into a group sends its requirement message $rm = (z^{r} ,ID)$ to the group manager, and subsequently obtains a valid AIA $\Phi = z^{r} \cdot H(ID) + \alpha \varepsilon \, \bmod p$ distributed by the group manager. The distribution of the AIA has been detailed discussed in Section "Authorization invisible authenticator".
- The group manager adds the related information into the UIT and updates the number of current users in the group, namely, *UN* = *UN* + 1.

**(3) KeyGen**

**Figure 3:**
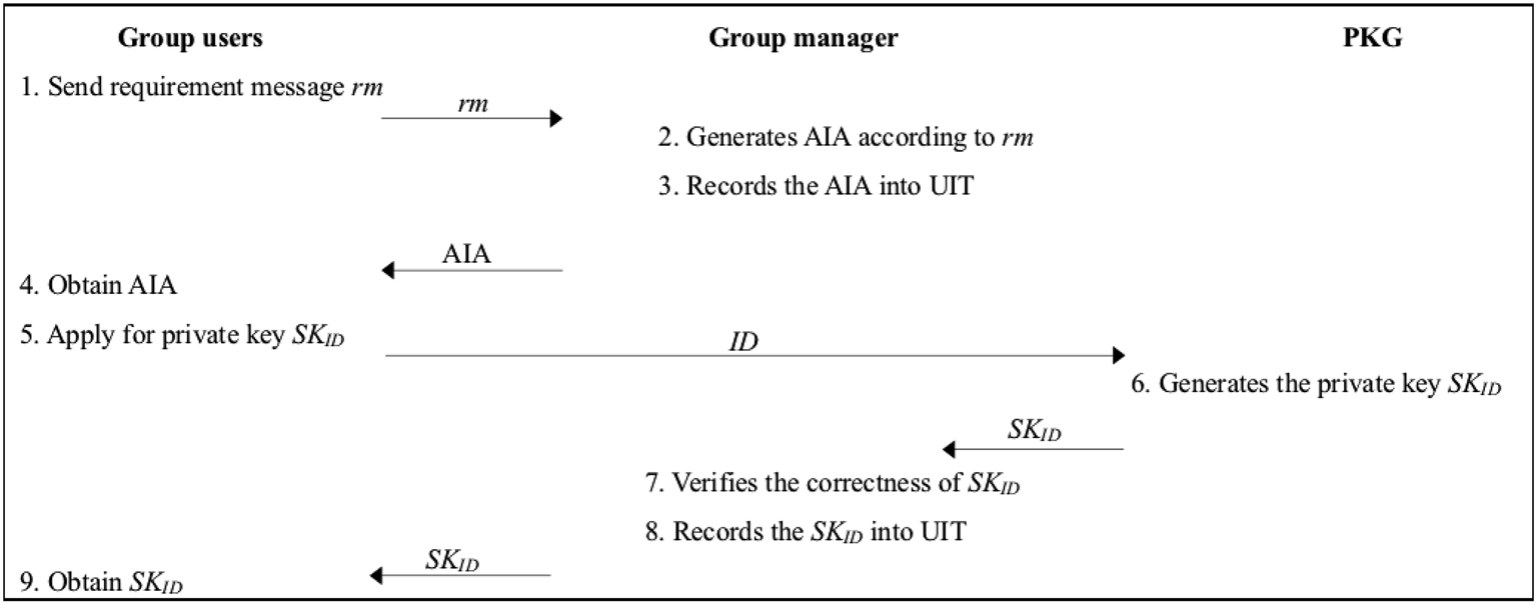
The process of applying for AIA and private key.

After receiving the key generation request from the group user, the PKG performs the following operations:

- The PKG picks $r_{ID} \in Z_{P}^{*}$ and computes $R_{ID} = g^{{r_{ID} }}$.
- The PKG calculates $R_{SK} = r_{ID} + x_{ID} H_{1} (ID)$. And then the PKG returns $SK_{ID} = (R_{ID} ,R_{SK} )$ to the group manager.
- After receiving the private key *SK*_*ID*_, the group manager verifies its correctness according to (3):
  3 $$ g^{{R_{SK} }} \mathop = \limits^{?} R_{ID} (mpk_{ID} )^{{H_{1} (ID)}} $$
  If (3) holds, the group manager accepts it and adds it into the UIT. Otherwise, reject it and inform to retransmit.
- The group manager forwards the *SK*_*ID*_ to the group user. The specific process of the user applying for AIA and the private key is shown in Fig. 3.

**(4) TagGen**

For the group users, the calculation process of the shared data tags set is depicted as follows:

- The group user divides shared file *F* into *n* blocks $F = \{ b_{i} \}_{1 \le i \le n}$, where $b_{i} \in Z_{P}^{*}$.
- The group user sets the $\varpi = H_{2} (name||n)$, where $name \in Z_{P}^{*}$ is a random value selected as the file identifier.
- For block $b_{i}$, the group user computes $T = \{ \sigma_{i} \}_{1 \le i \le n}$ , where $\sigma_{i} = (\varpi \cdot \mu^{{b_{i} }} )^{{R_{SK} }}$.
- The group user sends shared file and its corresponding tags set {*F*, *T*} to the group manager, and deletes the local storage. Subsequently, the group manager performs the data upload work.

**(5) UpdateForUsers**

When users are revoked from the group, the group manager executes the **AuthUpdate** algorithm to recompute and redistribute AIAs for the rest of group users, resets *UN* = *UN* -1, and then updates the UIT (the detailed operations are mentioned in Section "Authorization invisible authenticator").

**(6) Challenge**

After obtaining the group user authorization, the TPA periodically performs remote data integrity checking, records audit results, and regularly feeds back it to the group manager. The public auditing process for shared data is as follows:

- The TPA determines a *c*, where $1 \le c \le n$.
- The TPA generates a PRP key $k_{1}$ and a PRF key $k_{2}$, where $k_{1} ,k_{2} \in Z_{P}^{*}$.
- The TPA sends $chal = \{ c,k_{1} ,k_{2} \}$ to the CSP.

**(7) ProofGen**

After the CSP receiving the inquiry message *chal*, it generates an audit proof and responds the TPA.

- The CSP computes $\{ i\} = \pi_{{k_{1} }} (l)_{1 \le c \le n} ,\{ v_{i} \} = f_{{k_{2} }} (l)_{1 \le c \le n}$.
- The CSP calculates $\lambda = \sum\nolimits_{{(i,v_{i} ) \in Q}} {v_{i} b_{i} }$, $\sigma = \prod\nolimits_{{(i,v_{i} ) \in Q}} {\sigma_{i}^{{v_{i} }} }$.
- The CSP sends the audit proof $P = \{ \lambda ,\sigma \}$ to the TPA.

**(8) ProofVerify**

After received the audit proof, the TPA checks its correctness through Eq. (4):

4 $$ e(\sigma ,g)\mathop = \limits^{?} e\left( {\prod\nolimits_{{(i,v_{i} ) \in Q}} {\varpi^{{v_{i} }} } \cdot \mu^{\lambda } ,R_{ID} (mpk_{ID} )^{{H_{1} (ID)}} } \right) $$

If (4) holds, returns “1”, which means that the shared data stored in the CSP is intact; Otherwise, returns “0”. The TPA carefully and accurately records the results of each audit, and regularly feeds back it to the group manager.

## Security analysis

In this section, we prove that the proposed scheme is secure in term of the correctness and audit soundness.

### **Theorem 5**

(Correctness). *Our proposed scheme satisfies the following correctness*:

- **Private key correctness:** *A valid private key can be passed the correctness verification of the group manager*.
- **AIA correctness:** *A valid AIA* $\Phi$ *can be passed the verification of the group manager*.
- **Audit correctness:** *Only when the CSP stores all the shared data of the group user, during the public audit phase, the audit proof P he generates can pass the TPA’s correctness verification.*

### *Proof*

(a) Given a private key *SK*_*ID*_ generated by the PKG, the group manager can verify whether the left and right sides of (3) are equal to determine the availability of the *SK*_*ID*_.

$$ g^{{R_{SK} }} = g^{{r_{ID} + x_{ID} H_{1} (ID)}} = g^{{r_{ID} }} g^{{x_{ID} H_{1} (ID)}} = g^{{r_{ID} }} g^{{(x_{ID} )H_{1} (ID)}} = R_{ID} (mpk_{ID} )^{{H_{1} (ID)}} $$

(b) The AIA’s correctness proof has been given in Section "Authorization invisible authenticator".

(c) Given an audit proof P returned by the CSP, the TPA can verify whether the left and right sides of (4) are equal to determine the *P*’s correctness.

$$ \begin{aligned} e(\sigma ,g) & = e\left( {\prod\nolimits_{{(i,v_{i} ) \in Q}} {\sigma_{i}^{{v_{i} }} } ,g} \right) = e\left( {\prod\nolimits_{{(i,v_{i} ) \in Q}} {\left( {(\varpi \cdot \mu^{{b_{i} }} )^{{R_{SK} }} } \right)^{{v_{i} }} } ,g} \right) \\ & = e\left( {\prod\nolimits_{{(i,v_{i} ) \in Q}} {\left( {\varpi \cdot \mu^{{b_{i} }} } \right)}^{{v_{i} }} ,g^{{R_{SK} }} } \right) = e\left( {\prod\nolimits_{{(i,v_{i} ) \in Q}} {\varpi^{{v_{i} }} } \cdot \prod\nolimits_{{(i,v_{i} ) \in Q}} {\mu^{{b_{i} v_{i} }} ,g^{{r_{ID} + x_{ID} H_{1} (ID)}} } } \right) \\ & = e\left( {\prod\nolimits_{{(i,v_{i} ) \in Q}} {\varpi^{{v_{i} }} } \cdot \mu^{{\sum\nolimits_{{(i,v_{i} ) \in Q}} {b_{i} v_{i} } }} ,R_{ID} (mpk_{ID} )^{{H_{1} (ID)}} } \right) = e\left( {\prod\nolimits_{{(i,v_{i} ) \in Q}} {\varpi^{{v_{i} }} } \cdot \mu^{\lambda } ,R_{ID} (mpk_{ID} )^{{H_{1} (ID)}} } \right) \\ \end{aligned} $$

If (4) holds, it indicates that the shared data stored in the CSP is intact, returns “1”; Otherwise, returns “0”.

### **Theorem 6**

(Audit soundness). *If the CDH assumption and DL assumption hold in* *G*_1_, *and the tags set is existentially unforgeable; then, the CSP cannot pass the TPA’s verification with negligible probability, in the case that it does not fully possess user’s intact file*.

### *Proof*

We construct a knowledge extractor to extract the challenged block through multiple interactions with the proposed scheme, which is same to reference^3^. That is, if the adversary $\mathcal{A}$ can pass the TPA’ verification but not possess the intact data, we can extract the challenged block by repeated interactions between the knowledge extractor and the proposed scheme. Here, we assume that the CSP acts as an adversary $\mathcal{A}$, and the TPA is regarded as the challenger $\mathcal{C}$. By executing the following series of games to implement repeated interactions between $\mathcal{A}$ and $\mathcal{C}$, the audit soundness of proposed scheme can be proved.

**Game 0:** The challenger $\mathcal{C}$ runs the **Setup** algorithm to obtain the system public parameter *pp* and master secret key *msk*, then sends *pp* to the adversary $\mathcal{A}$. Next, the adversary $\mathcal{A}$ makes two types of queries respectively: (1) The adversary $\mathcal{A}$ queries the user’s private key. The challenger $\mathcal{C}$ runs the ***KeyGen*** algorithm to obtain and send the private key *SK*_*ID*_ to the adversary $\mathcal{A}$. (2) The adversary $\mathcal{A}$ queries the tags set of the shared file *F*. The challenger $\mathcal{C}$ runs the ***TagGen*** algorithm to obtain and send the tags set *T* of the *F* to the adversary $\mathcal{A}$. Then, the challenger $\mathcal{C}$ sends the integrity challenge *chal* to the adversary $\mathcal{A}$ and request for audit proof P. Upon received *chal*, the adversary $\mathcal{A}$ computes and replies to the audit proof P. If the audit proof P can pass the verification of the challenger $\mathcal{C}$ with non-negligible probability, we say that the adversary $\mathcal{A}$ wins this game.

**Game 1: Game 1** is the same as **Game 0**, but there exists a minor difference. The challenger $\mathcal{C}$ keeps a list with recording all tags that the adversary $\mathcal{A}$ has ever queried. Whenever the adversary $\mathcal{A}$ makes the **TagGen** query, the challenger $\mathcal{C}$ adds a record into this list.

**Game 2: Game 2** is the same as **Game 1**, but there exists a minor difference. The challenger $\mathcal{C}$ keeps a list with recording all responses that the adversary $\mathcal{A}$ has ever responded.

Consider the following situation: the adversary $\mathcal{A}$ has passed the challenger $\mathcal{C}$’s correctness verification, but he does not hold the intact challenged blocks. Once this occurs, we say that the adversary $\mathcal{A}$ has won, but the game will be aborted.

According to the above description, we know that if the response $P = \{ \lambda ,\sigma \}$ has passed the challenger $\mathcal{C}$’s verification, the *P* must pass the verification of (5).

5 $$ e(\sigma ,g) = e\left( {\prod\nolimits_{{(i,v_{i} ) \in Q}} {\varpi^{{v_{i} }} } \cdot \mu^{\lambda } ,R_{ID} (mpk_{ID} )^{{H_{1} (ID)}} } \right) $$

Assume that the forged proof is $P^{\prime } = \{ \lambda^{\prime } ,\sigma^{\prime } \}$. Note that since the adversary $\mathcal{A}$ has won, then, we have $P = P^{\prime }$, but the game will be aborted. At this point, there are two situations: $\sigma \ne \sigma^{\prime } ,\lambda = \lambda^{\prime }$ or $\sigma = \sigma^{\prime } ,\lambda \ne \lambda^{\prime }$ and we analyze separately.

(a) If the aggregated tags $\sigma \ne \sigma^{\prime }$, that is, $\sigma^{\prime } = \prod\nolimits_{{(i,v_{i} ) \in Q}} {\sigma_{i}^{{\prime v_{i} }} }$ is not equal $\sigma$. since the returned response $P^{\prime } = \{ \lambda^{\prime } ,\sigma^{\prime } \}$ has successfully passed the correctness verification, the game will be aborted.

**Analysis**: The forged proof is $P^{\prime } = \{ \lambda^{\prime } ,\sigma^{\prime } \}$. Because the forgery is successful, we have the following formula holds.

6 $$ e\left( {\sigma^{\prime } ,g} \right) = e\left( {\prod\nolimits_{{(i,v_{i} ) \in Q}} {\varpi^{{v_{i} }} } \cdot \mu^{{\lambda^{\prime}}} ,R_{ID} (mpk_{ID} )^{{H_{1} (ID)}} } \right) $$

Now we construct a simulator which can break CDH assumption with the non-negligible probability, in case that the adversary $\mathcal{A}$ makes the game aborted. The goal of the simulator is to output $h^{\alpha }$ when $g,g^{\alpha } ,h$ as input. The simulator acts like the challenger $\mathcal{C}$ in **Game** 1 but has some difference.

The simulator chooses two random values $a,b \in Z_{P}^{*}$ and set $\mu = g^{a} h^{b}$. Then, the simulator interacts with the adversary $\mathcal{A}$. When received the forged proof $P^{\prime } = \{ \lambda^{\prime } ,\sigma^{\prime } \}$, the simulator checks whether (6) holds. Obviously, $\lambda^{\prime } \ne \lambda$; otherwise, $\sigma^{\prime } = \sigma$. We define $\Delta \lambda = \lambda^{\prime } - \lambda$. By dividing (6) by (5), setting the public value $mpk_{ID} = g^{\alpha }$, we get:

$$ \begin{aligned} e\left( {\sigma^{\prime } /\sigma ,g} \right) & = e\left( {\mu^{\Delta \lambda } ,R_{ID} (mpk_{ID} )^{{H_{1} (ID)}} } \right) = e\left( {(g^{a} h^{b} )^{\Delta \lambda } ,g^{{R_{ID} }} (mpk_{ID} )^{{H_{1} (ID)}} } \right) \\ & = e\left( {g^{a\Delta \lambda } ,g^{{R_{ID} }} (mpk_{ID} )^{{H_{1} (ID)}} } \right) \cdot e\left( {h^{b\Delta \lambda } ,g^{{R_{ID} }} (mpk_{ID} )^{{H_{1} (ID)}} } \right) \\ & = e\left( {g^{{a\Delta \lambda R_{ID} }} \cdot (mpk_{ID} )^{{a\Delta \lambda H_{1} (ID)}} ,g} \right) \cdot e(h^{{b\Delta \lambda R_{ID} }} ,g) \cdot e\left( {h^{b\Delta \lambda } ,(mpk_{ID} )^{{H_{1} (ID)}} } \right) \\ & = e\left( {g^{{a\Delta \lambda R_{ID} }} \cdot (mpk_{ID} )^{{a\Delta \lambda H_{1} (ID)}} \cdot h^{{b\Delta \lambda R_{ID} }} ,g} \right) \cdot e\left( {h^{b\Delta \lambda } ,(mpk_{ID} )^{{H_{1} (ID)}} } \right) \\ \end{aligned} $$

Next, we have

$$ \begin{aligned} & e\left( {\sigma^{\prime } \cdot \sigma^{ - 1} \cdot g^{{ - a\Delta \lambda R_{ID} }} \cdot (mpk_{ID} )^{{ - a\Delta \lambda H_{1} (ID)}} \cdot h^{{ - b\Delta \lambda R_{ID} }} ,g} \right) \\ & \quad = e\left( {h^{b\Delta \lambda } ,(mpk_{ID} )^{{H_{1} (ID)}} } \right) = e(h,mpk_{ID} )^{{b\Delta \lambda H_{1} (ID)}} = e(h,g^{\alpha } )^{{b\Delta \lambda H_{1} (ID)}} = e(h^{\alpha } ,g)^{{b\Delta \lambda H_{1} (ID)}} \\ \end{aligned} $$

Further, we can obtain $h^{\alpha } = \left( {\sigma^{\prime } \cdot \sigma^{ - 1} \cdot g^{{ - a\Delta \lambda R_{ID} }} \cdot (mpk_{ID} )^{{ - a\Delta \lambda H_{1} (ID)}} \cdot h^{{ - b\Delta \lambda R_{ID} }} ,g} \right)^{{{1 \mathord{\left/ {\vphantom {1 {b\Delta \lambda H_{1} (ID)}}} \right. \kern-0pt} {b\Delta \lambda H_{1} (ID)}}}}$. If the CDH problem can be solved in *G*_*1*_, then $h^{\alpha }$ must be calculable. Clearly, the probability of solving the CDH problem in *G*_*1*_ is equivalent to compute the probability of $b\Delta \lambda H_{1} (ID) = 0$. The probability of $b\Delta \lambda H_{1} (ID) = 0$ is 1/*p* which is negligible since *p* is a large prime. So, the probability of solving the CDH problem in *G*_*1*_ is 1 − 1/*p*. Further, the probability of the game abort is 1 − 1/*p*. Hence, if there is a non-negligible difference between the adversary $\mathcal{A}$’s probabilities of success in **Game** 1 and **Game** 2, then the constructed simulator can solve the CDH problem.

(b) If the aggregated block $\lambda \ne \lambda^{\prime }$, but the returned response $P^{\prime } = \{ \lambda^{\prime } ,\sigma^{\prime } \}$ has successfully passed the correctness verification, the game will be aborted.

**Analysis**: We also construct a simulator can break DL assumption with the non- negligible probability, in case that the adversary $\mathcal{A}$ makes the game aborted. The goal of the simulator is to output $\alpha$ when $g,h = g^{\alpha }$ as input. The simulator acts like the challenger $\mathcal{C}$ in **Game** 1 but has some difference.

The simulator also chooses two random values $a,b \in Z_{P}^{*}$ and set $\mu = g^{a} h^{b}$. Here, we have $\sigma^{\prime } = \sigma$ but $\lambda^{\prime } \ne \lambda$. Then we define $\Delta \lambda = \lambda^{\prime } - \lambda$. By dividing (6) by (5), we can obtain:

$$ 1 = \mu^{\Delta \lambda } = (g^{a} h^{b} )^{\Delta \lambda } = g^{a\Delta \lambda } \cdot h^{b\Delta \lambda } $$

Obviously, $\Delta \lambda \ne 0\bmod p$; otherwise, $\lambda^{\prime } = \lambda \bmod p$ which contradicts after mentioned assumption.

Further we have $h = g^{{{{ - a\Delta \lambda } \mathord{\left/ {\vphantom {{ - a\Delta \lambda } {b\Delta \lambda }}} \right. \kern-0pt} {b\Delta \lambda }}}} = g^{{{a \mathord{\left/ {\vphantom {a b}} \right. \kern-0pt} b}}}$. So, the probability of solving the DL problem in *G*_*1*_ is equivalent to compute the probability of *b* = 0. The probability of *b* = 0 is 1/*p* which is negligible since *p* is a large prime. So, the probability of solving the DL problem in *G*_*1*_ is 1–1/*p*. Further, the probability of the game abort is 1–1/*p*. Hence, if there is a non- negligible difference between the adversary $\mathcal{A}$’s probabilities of success in **Game** 1 and **Game** 2, then the constructed simulator can solve the DL problem.

## Performance evaluation

In this section, we first give the functionality comparison between our scheme and other related schemes, show the computation and communication overheads at different phases, and then conduct performance evaluation through several experiments.

### Functionality comparison

We discuss the functionality comparison between our scheme and scheme^3,24,29,30^ in terms of public verification, group authority management and user revocation. The results are shown as Table 3. Note that our scheme is the only one that satisfies those three properties. Moreover, none of the schemes^3,24,29,30^ can support group authority management.

**Table 3:** Functionality comparison with other related schemes.

Schemes	Public verification	Group authority management	User revocation
Shacham et al.^3^	Yes	No	No
Wang et al.^24^	Yes	No	Yes
Zhang et al.^29^	Yes	No	Yes
Fu et al.^30^	Yes	No	Yes
Our scheme	Yes	Yes	Yes

### Performance analysis and comparison

For simplicity, we give the meaning of the following notations firstly. Here $Hash_{{G_{1} }} ,Hash_{{Z_{P}^{*} }}$ denote the hash operation in $G_{1} ,Z_{P}^{*}$. $Exp_{{G_{1} }} ,Exp_{{G_{T} }} ,Exp_{{Z_{P}^{*} }}$ express the exponentiation operation in $G_{1} ,G_{T} ,Z_{P}^{*}$.$Mul_{{G_{1} }} ,Mul_{{G_{T} }} ,Mul_{{Z_{P}^{*} }}$ mean the multiplication operation in $G_{1} ,G_{T} ,Z_{P}^{*}$. $Pair$ represents the pairing operation. $Add_{{G_{1} }} ,Add_{{Z_{P}^{*} }}$ indicate the addition operation in $G_{1} ,Z_{P}^{*}$. *c* indicates the number of the challenged blocks and *n* is the total number of data blocks. |*n*| is the size of an element of set [1, *n*]. |*p*| is the size of an element in $Z_{P}^{*}$, and |*q*| is the size of an element in $G_{1}$.

**(1) Performance analysis**

Tables 4 and 5 show the computation and communication overheads at different phases.

- Computation overhead. The Table 4 reveals the computation overheads at different phases. In the apply for authorization phase, user who applies to join the group need to generate a requirement message *rm*, and the computation overhead is $$Exp_{{Z_{P}^{*} }}$$. In the AIA generation phase, the group manager generates AIA for user according to its *rm*, and the computation overhead is $$Hash_{{Z_{P}^{*} }} + Mul_{{Z_{P}^{*} }} + Exp_{{Z_{P}^{*} }} + Add_{{Z_{P}^{*} }}$$. In the AIA update phase, the group manager needs to reselect random elements to get the new $$z^{r}$$, and the spending is the same as the AIA generation phase. The computation overhead of proof generation phase is $$cExp_{{G_{1} }} + (c - 1)Mul_{{G_{1} }} + cMul_{{Z_{P}^{*} }} +$$$$(c - 1)Add_{{Z_{P}^{*} }}$$. To perform the integrity verification, the TPA needs to check the correctness of (4). For computing $$\prod\nolimits_{{(i,v_{i} ) \in Q}} {\varpi^{{v_{i} }} } \cdot \mu^{\lambda }$$, our scheme requires (*c* + 1) exponentiation operations, 2 multiplicative operations, (*c* − 1) addition operation and *c* hash operations. And the cost of calculating $$R_{ID} (mpk_{ID} )^{{H_{1} (ID)}}$$ is $$Exp_{{G_{1} }} + Mul_{{G_{1} }} + Hash_{{Z_{P}^{*} }}$$.
- Communication overhead. The Table 5 reveals the communication overheads at different phases. In the apply for authorization phase, the communication overhead comes from transmitting *rm*. To distribute the AIA for every group user, the cost is the same and all requires $|p|$ bits in the AIA generation and AIA update phases. In our proposed scheme, the communication overhead is mainly from the public auditing process. The TPA sends $chal = \{ c,k_{1} ,k_{2} \}$ to the CSP, and the size of auditing challenge is $|p| + \log_{2} c$ bits. Then the CSP compute and reply the TPA, and the size of an audit proof $P = \{ \lambda ,\sigma \}$ is $|p| + |q|$ bits.

**Table 4:** The computation overhead of a group user at different phases.

Phase	Computation overhead
Apply for authorization	$Exp_{{Z_{P}^{*} }}$
AIA generation	$Hash_{{Z_{P}^{*} }} + Mul_{{Z_{P}^{*} }} + Exp_{{Z_{P}^{*} }} + Add_{{Z_{P}^{*} }}$
AIA update	$Hash_{{Z_{P}^{*} }} + Mul_{{Z_{P}^{*} }} + Exp_{{Z_{P}^{*} }} + Add_{{Z_{P}^{*} }}$
Proof generation	$cExp_{{G_{1} }} + (c - 1)Mul_{{G_{1} }} + cMul_{{Z_{P}^{*} }} + (c - 1)Add_{{Z_{P}^{*} }}$
Proof verification	$2Pair + (c + 2)Exp_{{G_{1} }} + 2Mul_{{G_{1} }} + (c - 1)Add_{{G_{1} }} + cHash_{{G_{1} }} + Hash_{{Z_{P}^{*} }}$

**Table 5:** The communication overhead of a group user at different phases.

Phase	Communication overhead
Apply for authorization	$2|p|$
AIA generation	$|p|$
AIA update	$|p|$
Challenge	$|p| + \log_{2} c$
Proof generation	$|p| + |q|$

**(2) Performance comparison**

Table 6 compares the computation overhead in terms of proof generation and proof verification algorithms with Wang et al.’s scheme^24^, Zhang et al.’s scheme^29^ and Fu et al.’s scheme^30^. From the Table 6, we know that all schemes have the same computation overhead at the proof generation phase. Since both schemes^24^ and^30^ are deployed in asymmetric bilinear pairings, only the comparison results are shown, and no further details will be given. In addition, the computational cost of our proposed scheme is lower than that of the scheme^29^.

**Table 6:** Comparison of the computation overhead at proof generation phase and proof verification phase in different schemes.

Scheme	Proof generation	Proof verification
Wang et al.^24^	$$\begin{gathered} cExp_{{G_{1} }} + (c - 1)Mul_{{G_{1} }} \hfill \\ + cMul_{{Z_{P}^{*} }} + (c - 1)Add_{{Z_{P}^{*} }} \hfill \\ \end{gathered}$$	$$\begin{gathered} 4Pair + 17cExp_{{G_{1} }} + 11cMul_{{G_{1} }} + 2cExp_{{Z_{P}^{*} }} \hfill \\ + (4c + k)Mul_{{Z_{P}^{*} }} + cExp_{{G_{T} }} + cMul_{{G_{T} }} \hfill \\ \end{gathered}$$
Zhang et al.^29^	$$\begin{gathered} cExp_{{G_{1} }} + (c - 1)Mul_{{G_{1} }} \hfill \\ + cMul_{{Z_{P}^{*} }} + (c - 1)Add_{{Z_{P}^{*} }} \hfill \\ \end{gathered}$$	$$\begin{gathered} 2Pair + (2c + 3)Exp_{{G_{1} }} + (2c + 2)Mul_{{G_{1} }} \hfill \\ + (c - 1)Add_{{Z_{P}^{*} }} + cMul_{{Z_{P}^{*} }} + cHash_{{G_{1} }} + 2Hash_{{Z_{P}^{*} }} \hfill \\ \end{gathered}$$
Fu et al.^30^	$$\begin{gathered} cExp_{{G_{1} }} + (c - 1)Mul_{{G_{1} }} \hfill \\ + cMul_{{Z_{P}^{*} }} + (c - 1)Add_{{Z_{P}^{*} }} \hfill \\ \end{gathered}$$	$2Pair + 2cExp_{{G_{1} }} + 2cMul_{{G_{1} }} + 22cExp_{{Z_{P}^{*} }} + 14cMul_{{Z_{P}^{*} }}$
Our scheme	$$\begin{gathered} cExp_{{G_{1} }} + (c - 1)Mul_{{G_{1} }} \hfill \\ + cMul_{{Z_{P}^{*} }} + (c - 1)Add_{{Z_{P}^{*} }} \hfill \\ \end{gathered}$$	$$\begin{gathered} 2Pair + (c + 2)Exp_{{G_{1} }} + 2Mul_{{G_{1} }} + (c - 1)Add_{{G_{1} }} \hfill \\ + cHash_{{G_{1} }} + Hash_{{Z_{P}^{*} }} \hfill \\ \end{gathered}$$

### Experimental results

In this subsection, we evaluate the performance of the proposed scheme by several experiments. We run a series of experiments on a 1.8 GHZ Intel Core i5 processor and 8 GB RAM. All the experiments using the Type A with the free Pairing-Based Cryptography (PBC) Library. We choose the based filed size to be 512 bits, and the size of $Z_{P}^{*}$ to be 160 bits, this is, |*p*|= 160 bits. We set the file size to 20 MB which divided into 1,000,000 data blocks.

**(1) Standard AIA generation phase**

**Figure 4:**
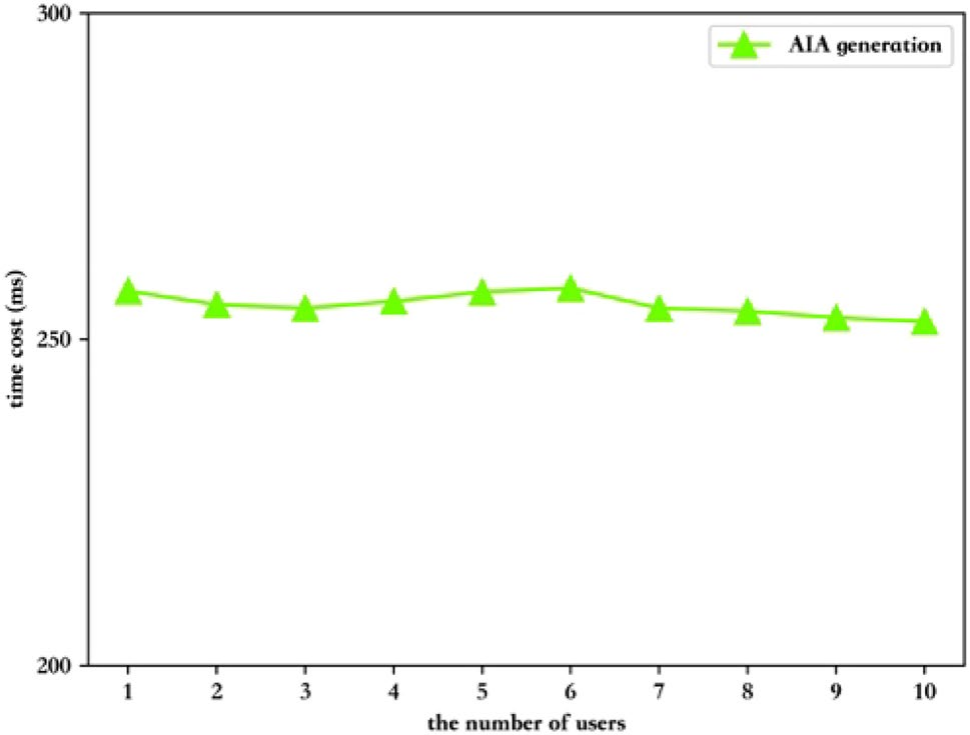
The time cost of AIA generation.

In the first part, we evaluate the computation cost of AIA generation phase. Figure 4 shows that as the number of users increases, the time to calculate AIA remains almost unchanged, and it is parallel to the *x*-axis, which takes about 260 ms. The reason is that the AIA generated by the group manager for the user is calculated separately, and the overhead of calculation required is small.

**(2) Auditing phase**

**Figure 5:**
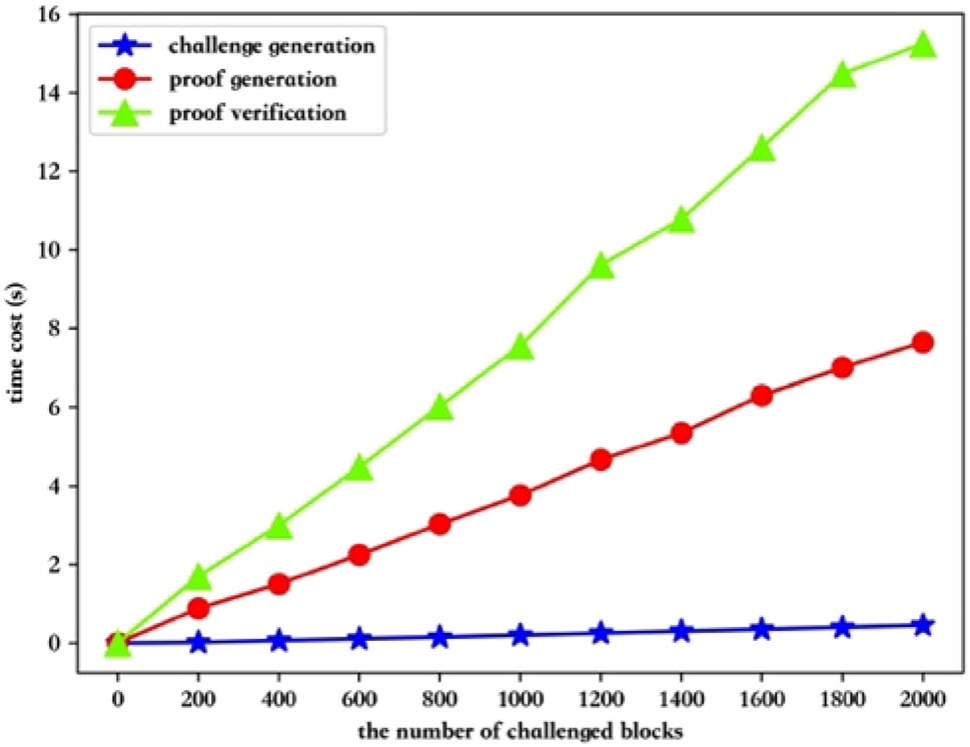
The time cost in the public auditing process.

The public auditing process divide into launch integrity challenge, proof generation and proof verification. To evaluate the performance of the auditing process, we set the number of challenged blocks from 0 to 2000, with 200 as the interval. Figure 5 shows that the time cost in the three phases is directly proportional to the number of data blocks to be challenged. That is, as the number of challenged data blocks increases, the time cost increases. The launch integrity challenge takes the least amount of time, ranging from 0.015840 to 0.450362 s. When the number of challenged data blocks is 200 and 2000, the proof generation time is 0.882022 s and 7.645638 s respectively. The proof verification phase is the most time-consuming, with the time varying from 1.698569 to 15.251921 s.

**(3) Proof verification phase**

**Figure 6:**
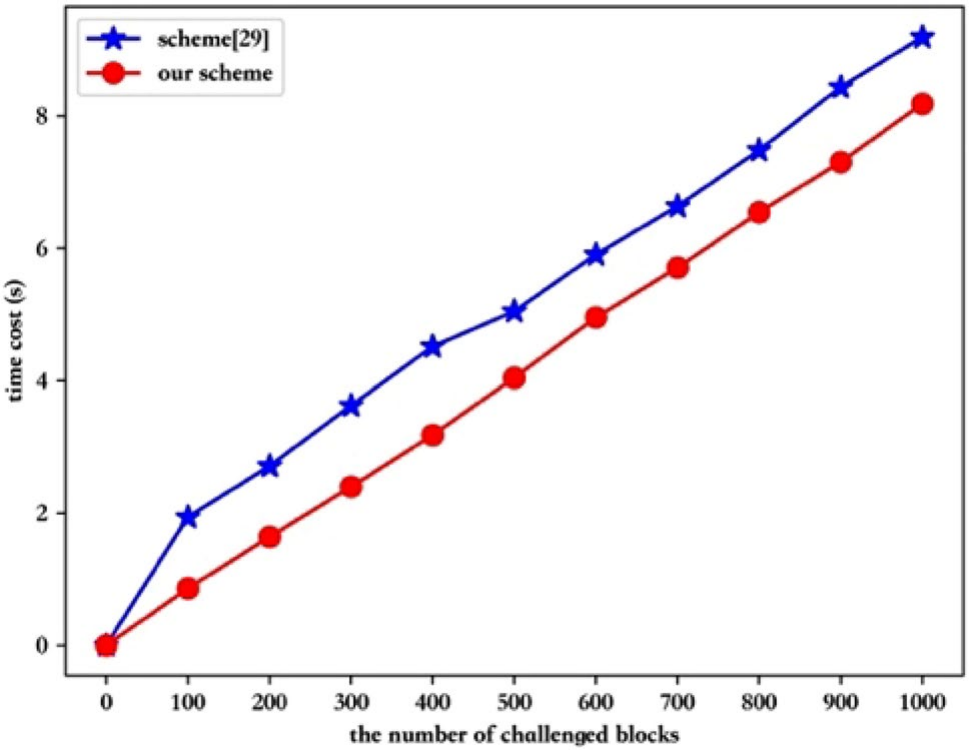
Comparison in the proof verification phase.

In the third part, we evaluate the performance of proof verification phase through comparing our scheme with Zhang et al.’s scheme^29^. We set the number of the challenged blocks from 0 to 1000, increased by an interval of 100. Figure 6 gives more details. Clearly, our scheme spends less than Zhang et al.’s scheme^29^, which is consistent with the results obtained by the previous computational overhead analysis. As the number of challenged data blocks increases, the time cost in the proof verification phase of two schemes increases linearly. And our scheme time ranged from 0.857812 to 8.184904 s, while the Zhang et al.’s scheme^29^ varied from 1.939095 to 9.195359 s.

## Conclusion

In this paper, we propose a remote data possession checking scheme that supports group user authorization management in public cloud storage environments. To realize group user authority management, a trusted entity named group manager is introduced. Furthermore, a standard AIA scheme is constructed to assist group manager to perform rights management, and a detailed algorithm and security analysis are given. After solving the problem of authority management, we propose an integrity verification scheme for shared data. The scheme is designed based on IBE, which avoids the cost of certificate management caused by key escrow, and the security analysis and a series of performance evaluations show that it is safe and feasible.

## Data Availability

Data sharing not applicable to this paper as no datasets were generated or analyzed during the current study.
